# DNase I and chitosan enhance efficacy of ceftazidime to eradicate *Burkholderia pseudomallei* biofilm cells

**DOI:** 10.1038/s41598-023-27790-2

**Published:** 2023-01-19

**Authors:** Rattiyaphorn Pakkulnan, Nuttaya Thonglao, Sorujsiri Chareonsudjai

**Affiliations:** 1 Department of Microbiology, Faculty of Medicine, Khon Kaen University, Khon Kaen, Thailand; 2 Research and Diagnostic Center for Emerging Infectious Diseases (RCEID), Khon Kaen University, Khon Kaen, Thailand

**Keywords:** Chemical biology, Microbiology

## Abstract

Biofilm-associated *Burkholderia pseudomallei* infection contributes to antibiotic resistance and relapse of melioidosis. *Burkholderia pseudomallei* biofilm matrix contains extracellular DNA (eDNA) that is crucial for biofilm establishment. However, the contribution of eDNA to antibiotic resistance by *B. pseudomallei* remains unclear. In this study, we first demonstrated in vitro that DNase I with the administration of ceftazidime (CAZ) at 24 h considerably inhibited the 2-day biofilm formation and reduced the number of viable biofilm cells of clinical *B. pseudomallei* isolates compared to biofilm treated with CAZ alone. A 3–4 log reduction in numbers of viable cells embedded in the 2-day biofilm was observed when CAZ was combined with DNase I. Confocal laser-scanning microscope visualization emphasized the competence of DNase I followed by CAZ supplementation to significantly limit *B. pseudomallei* biofilm development and to eradicate viable embedded *B. pseudomallei* biofilm cells. Furthermore, DNase I supplemented with chitosan (CS) linked with CAZ (CS/CAZ) significantly eradicated shedding planktonic and biofilm cells. These findings indicated that DNase I effectively degraded eDNA leading to biofilm inhibition and dispersion, subsequently allowing CAZ and CS/CAZ to eradicate both shedding planktonic and embedded biofilm cells. These findings provide efficient strategies to interrupt biofilm formation and improve antibiotic susceptibility of biofilm-associated infections.

## Introduction

Melioidosis, a deadly infectious disease caused by *Burkholderia pseudomallei* was discovered in 1911^1^ but still remains a significant public health concern worldwide^2^. A modeling study has forecast as many as 165,000 melioidosis cases with ~ 89,000 deaths per year worldwide^3^ and 2,800 deaths yearly in Thailand alone^4^. The mortality rate varies in endemic regions; 23% in Australia^5^, 39% in Thailand^6^ and up to 61% in Cambodia^7^. Early diagnosis, effective antimicrobial therapy and innovative intensive care can reduce mortality rates to less than 10%^8^. Melioidosis patients exhibit diverse clinical presentations and a wide range of severity. Relapsing melioidosis, due to persistence of the original infection as a result of inadequate treatment, leads to a high mortality rate^9–12^. Relapse occurs in approximately 10% of melioidosis patients^2^.

Therapeutic management of melioidosis comprises initial intravenous administration of ceftazidime (CAZ) for at least 10 days, followed by oral trimethoprim-sulfamethoxazole (TMP-SMX) for 12 to 20 weeks^13–15^. Antimicrobial resistance of clinical *B. pseudomallei* is infrequent^16^ but β-lactam resistance can be associated with the alteration of a gene encoding a penicillin-binding protein 3 during prolonged ceftazidime therapy^17^. Therefore, further investigations of new therapeutic strategies based on fundamental research are essential to reduce mortality in endemic areas and limit development of antibiotic resistance in *B. pseudomallei*.

The facultative intracellular behavior and possession of virulence factors, including biofilm-formation ability of *B. pseudomallei*, can facilitate the survival and persistence of this pathogen. Of particular importance is the ability to form biofilm: isolation of biofilm from the primary infection of relapsing melioidosis patients suggests a correlation with bacterial persistence^18^. Cells of *B. pseudomallei* find shelter within the biofilm matrix, as demonstrated in laboratory studies^19,20^ and in lung tissue of infected humans and animals^21^. Cells within biofilm have an intrinsic resistance to CAZ and TMP-SMX reinforced the lack of success in the treatment of the chronic manifestations melioidosis management. An in vitro study by Sawasdidoln and colleagues showed that *B. pseudomallei* biofilm formation is associated with resistance to doxycycline, CAZ, imipenem, and TMP-SMX: efflux pumps play a role in this^22^. The extracellular polymeric constituents of *B. pseudomallei* biofilm can limit penetration of antibiotics^23,24^. Biofilm associated bacteria are more resistant to antibiotics than free-living (planktonic) cells. *Burkholderia pseudomallei* biofilm forms were more tolerant of CAZ than were planktonic cells at the minimum biofilm eradication concentration of 2,048 µg/mL or more^25^. To eradicate persistent cells in biofilms, a combination of agents, including antibiotics, enzymes and antimicrobial agents, may be required^26^. Clearly, innovative therapeutic regimens are needed that can inhibit or disperse biofilm, exposing the shed planktonic cells to the full effects of antibiotics.

Extracellular DNA (eDNA) is a self-produced component of the biofilm matrix that necessary for both initial attachment of cells and early bacterial biofilm formation. It is also associated with antibiotic resistance. Aminoglycoside resistance of *Pseudomonas aeruginosa* biofilm cells was due to the binding of negatively charged eDNA to positively charged aminoglycosides^27^. The acidic milieu provided by accumulation of eDNA in the biofilm matrix served to promote aminoglycoside resistance in *B. pseudomallei*^28^. The ability of eDNA in *P. pseudomallei* biofilm to bind to metal cations contributed to decreased permeability of bacterial outer membrane to aminoglycosides. Therefore, the accumulation of eDNA in biofilm matrix contributes to the long-term survival of the pathogen^29^. The application of DNase I can degrade eDNA, resulting in interference with biofilm attachment and biofilm dispersion of *B. pseudomallei*^20^, *P. aeruginosa*^30^, *Listeria monocytogenes*^31,32^, and *Streptococcus mutans*^33^. Exposure to DNase assists in biofilm dispersal and prevention of biofilm formation of *P. aeruginosa* in cystic-fibrosis patients^30^. The greater susceptibility of liberated planktonic bacterial cells to ampicillin and ciprofloxacin has been demonstrated in DNase I-treated nontypeable *Hemophilus influenzae* biofilms^34^. Similarly, treatment of *Enterococcus faecalis* biofilms with DNase I efficiently sensitized the cells to 2% chlorhexidine^35^. Additionally, the combination of chitosan gel loaded with silver sulfadiazine, a frontline therapy in burn wound infections, supplemented with DNase I, was effective against *P. aeruginosa* biofilm-associated wound infections^36^. Chitosan (CS) was demonstrated to disrupt *B. pseudomallei* cell membrane resulted in intracellular constituents released and cell death^37^. Likewise, CS linked to CAZ was recently demonstrated to effectively kill *B. pseudomallei* biofilm cells^38^. Therefore, the elimination of eDNA, the principal component of the biofilm matrix supplemented with CS, a well-recognized antimicrobial and anti-biofilm biopolymer, has promise for biofilm disaggregation thus improving the bactericidal activity of CAZ against *B. pseudomallei* biofilm cells*.*

In this study, we have established that a combination of DNase I with either CAZ or CS/CAZ can improve the efficacy of CAZ against *B. pseudomallei* biofilm cells of three clinical isolates from lung, pus, and blood from melioidosis patients, *B. pseudomallei* L1, P1 and H777^20^. DNase I was hypothesized to be a biofilm-dispersant agent. In the present study, we provide evidence that DNase I contributes to *B. pseudomallei* biofilm inhibition and increases susceptibility of resident *B. pseudomallei* biofilm cells and shedding planktonic cells to CAZ and CS/CAZ.

## Results

### DNase I inhibits biofilm formation and enables CAZ to kill *B. pseudomallei* biofilm cells

**Figure 1:**
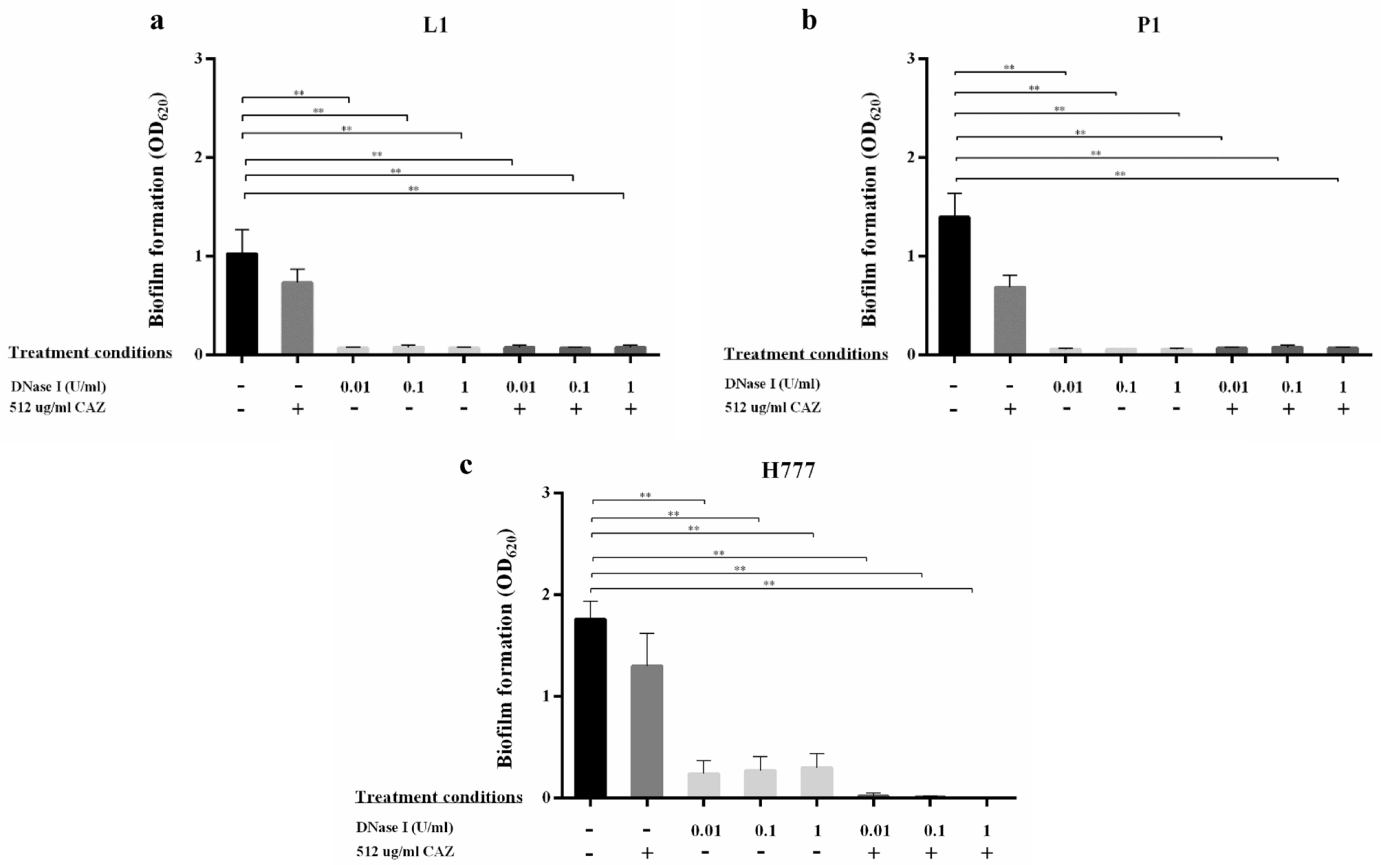
DNase I combined with ceftazidime inhibits *B. pseudomallei* biofilm formation. *Burkholderia pseudomallei* L1 (**a**), P1 (**b**) and H777 (**c**) were grown on pegs in 96-well plates in the presence of 0.01, 0.1 or 1 U/mL DNase I for the first 24 h. Subsequently, 512 µg/mL CAZ was added into the cultures for another 24 h. The 2-day biofilm formation was examined using crystal-violet staining. The experiment was performed in duplicate in each of three independent experiments. **p < 0.001.

**Figure 2:**
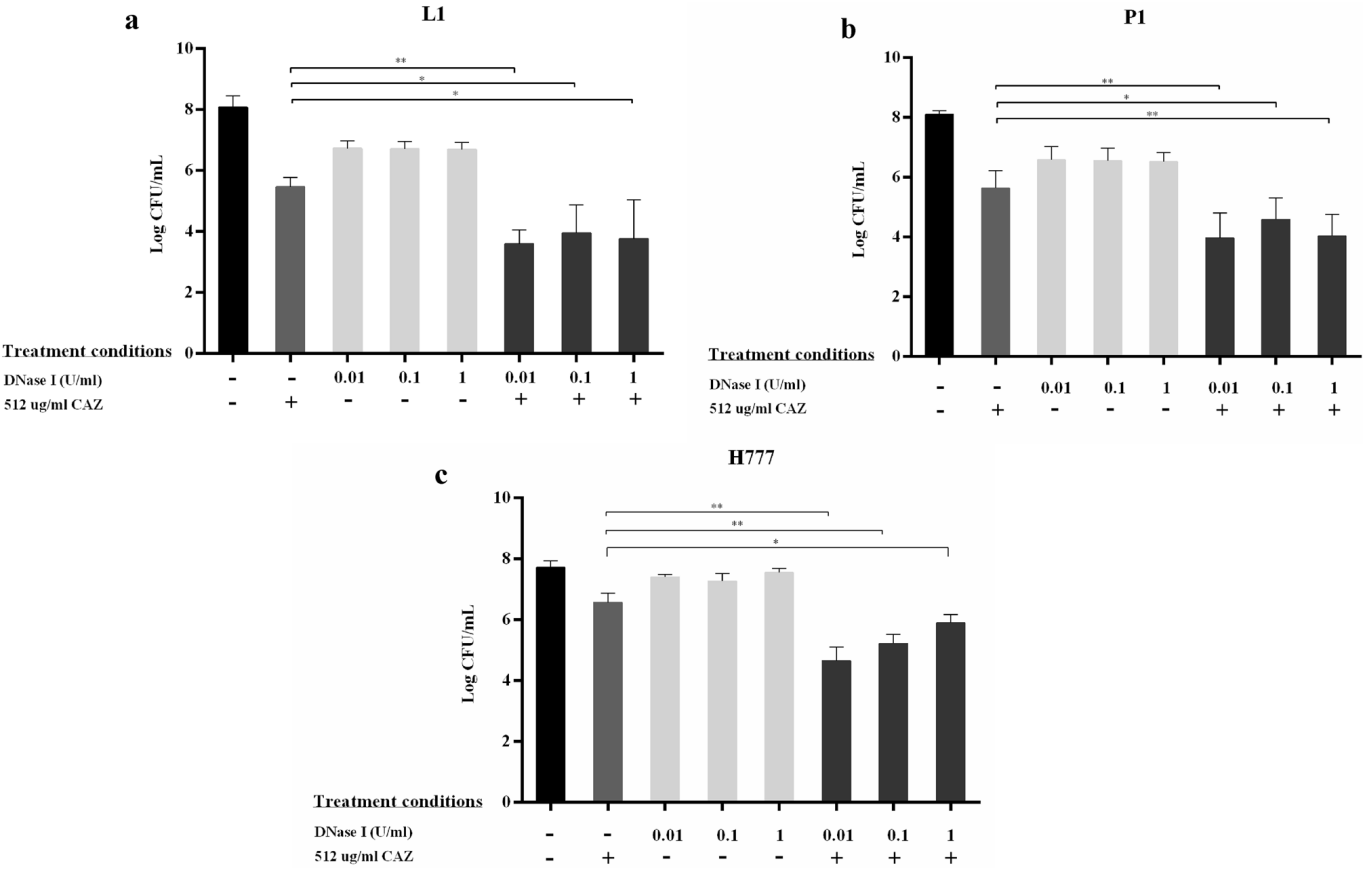
DNase I enhanced ceftazidime susceptibility during *B. pseudomallei* biofilm formation. *Burkholderia pseudomallei* L1 (**a**), P1 (**b**) and H777 (**c**) were grown on pegs in 96-well plates to form biofilm in the presence of 0.01, 0.1 or 1 U/mL DNase I for the first 24 h. Then, 512 µg/mL CAZ was added into the cultures for another 24 h. The 2-day viable biofilm cells were liberated by sonication for bacterial enumeration. The experiment was performed in duplicate in each of three independent experiments. *p < 0.05 and **p < 0.001.

Our previous report showed that DNase I interrupts *B. pseudomallei* biofilm adhesion and biofilm development^20^. We therefore hypothesized that the degradation of eDNA caused by DNase I interferes with the initial bacterial attachment step and interrupts biofilm formation. This, in turn, should increase CAZ susceptibility of *B. pseudomallei* cells. The first set of analyses examined the impact of the continuous presence of 0.01, 0.1 or 1 U/mL DNase I followed by adding 512 µg/mL CAZ at 24 h. DNase I alone interfered the biofilm formation as previously demonstrated^20^. The combination of DNase I with CAZ likewise significantly reduced biofilm formation compared to that of untreated control (*p* < 0.001 in each case) (Fig. 1). The same experiment remarkably improved efficiency of CAZ in eradication of viable *B. pseudomallei* biofilm cells compared to CAZ alone (*p* < 0.05 and *p* < 0.001) (Fig. 2). The most interesting aspect of these data is that only 0.01 U/mL DNase I was required to enable CAZ to bring about a 3–4 log reduction in numbers of *B. pseudomallei* biofilm cells compared to untreated controls. However, the bacterial susceptibility was not correlated to DNase I concentrations either alone or with CAZ. The DNase I-treated biofilm displayed the inefficiently stained with crystal violet while viable *B. pseudomallei* were detected. This finding may indicate the distinct biofilm biomass component after treated with DNase I that lack negatively charged biofilm components such as polysaccharides, proteins, or nucleic acids. While *B. pseudomallei* cells adhered to pegs indicating microcolonies of viable initial biofilms.

### DNase I degradation of eDNA resulted in *Burkholderia pseudomallei* biofilm inhibition and facilitated CAZ killing of embedded biofilm cells

**Figure 3:**
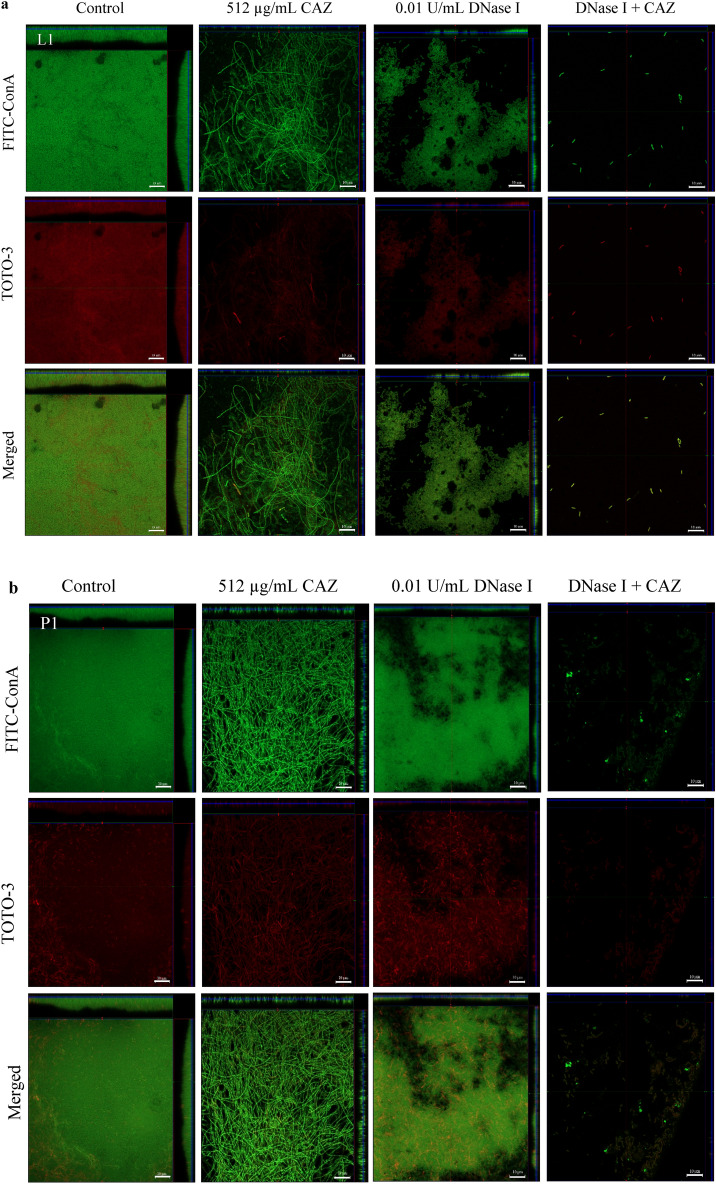

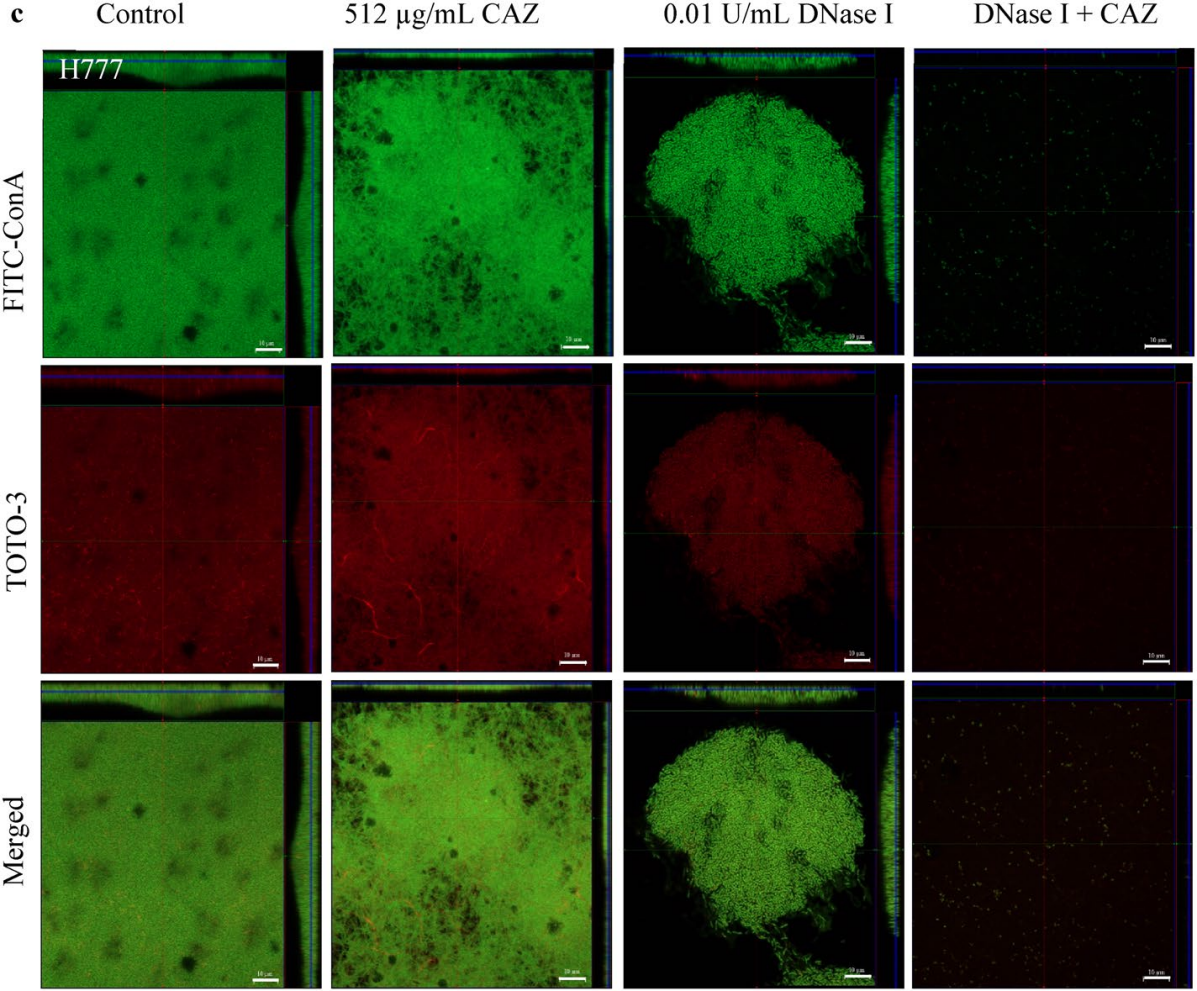
DNase I combined with CAZ eradicated *B. pseudomallei* L1, P1 and H777 biofilms. CLSM images of *B. pseudomallei* L1 (**a**), P1 (**b**) and H777 (**c**) biofilm biomass and eDNA grown on glass coverslips untreated (control) or treated with either 0.01 U/ml DNase I, or 512 µg/mL ceftazidime (CAZ), or DNase I + CAZ. The 2-day biofilms were then stained with FITC-ConA (biofilm biomass, green) and TOTO-3 (eDNA, red). The images are representative of three independent experiments and were taken using a Zeiss 800 CLSM microscope (63 × magnification). The scale bar represents 10 µm.

CLSM images of biofilms grown for 48 h in the presence of 0.01 U/mL DNase I with or without the addition of 512 µg/mL CAZ at 24 h are shown in Fig. 3. A change in morphology of biofilm cells from rod-shaped to filaments and clumps was observed in all three *B. pseudomallei* strains after treatment with CAZ alone. DNase I-treated biofilms revealed looser biofilm structures and the faint biofilm cells when treated with the combination of CAZ and DNase I.

### Live/dead visualization and live/dead ratio of *B. pseudomallei* biofilm cells treated with DNase I combined with CAZ

**Figure 4:**
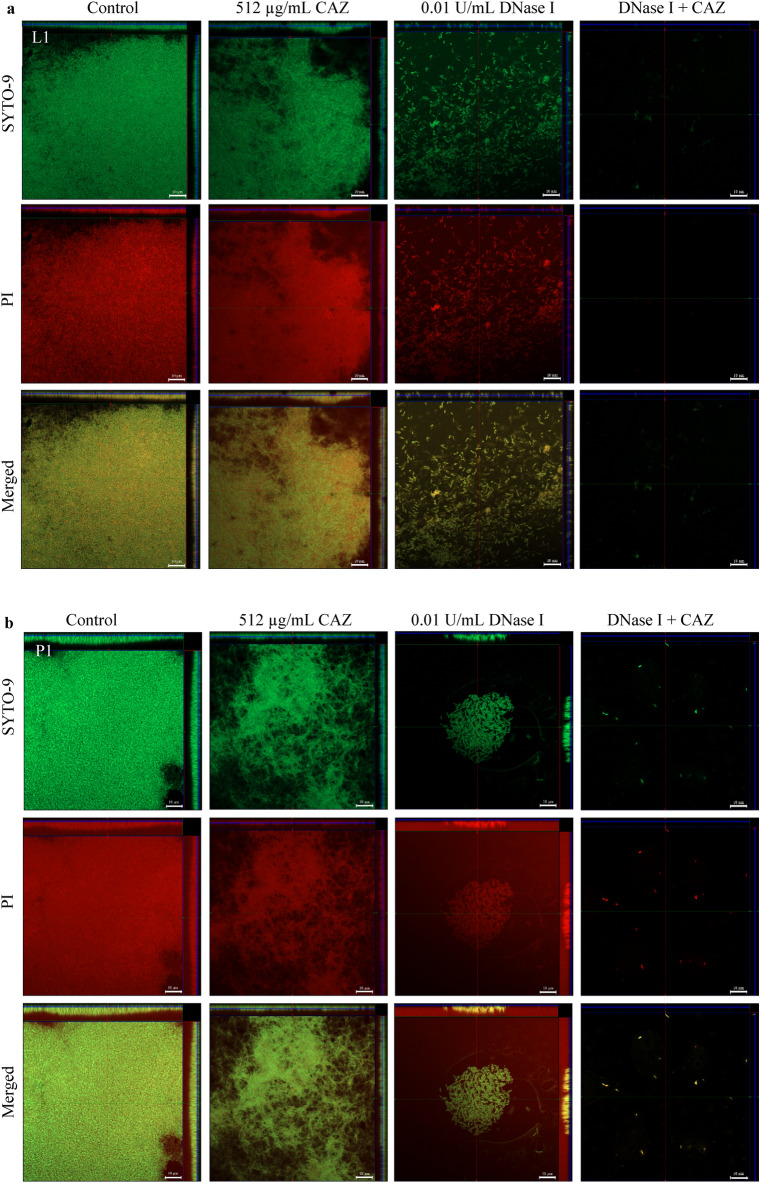

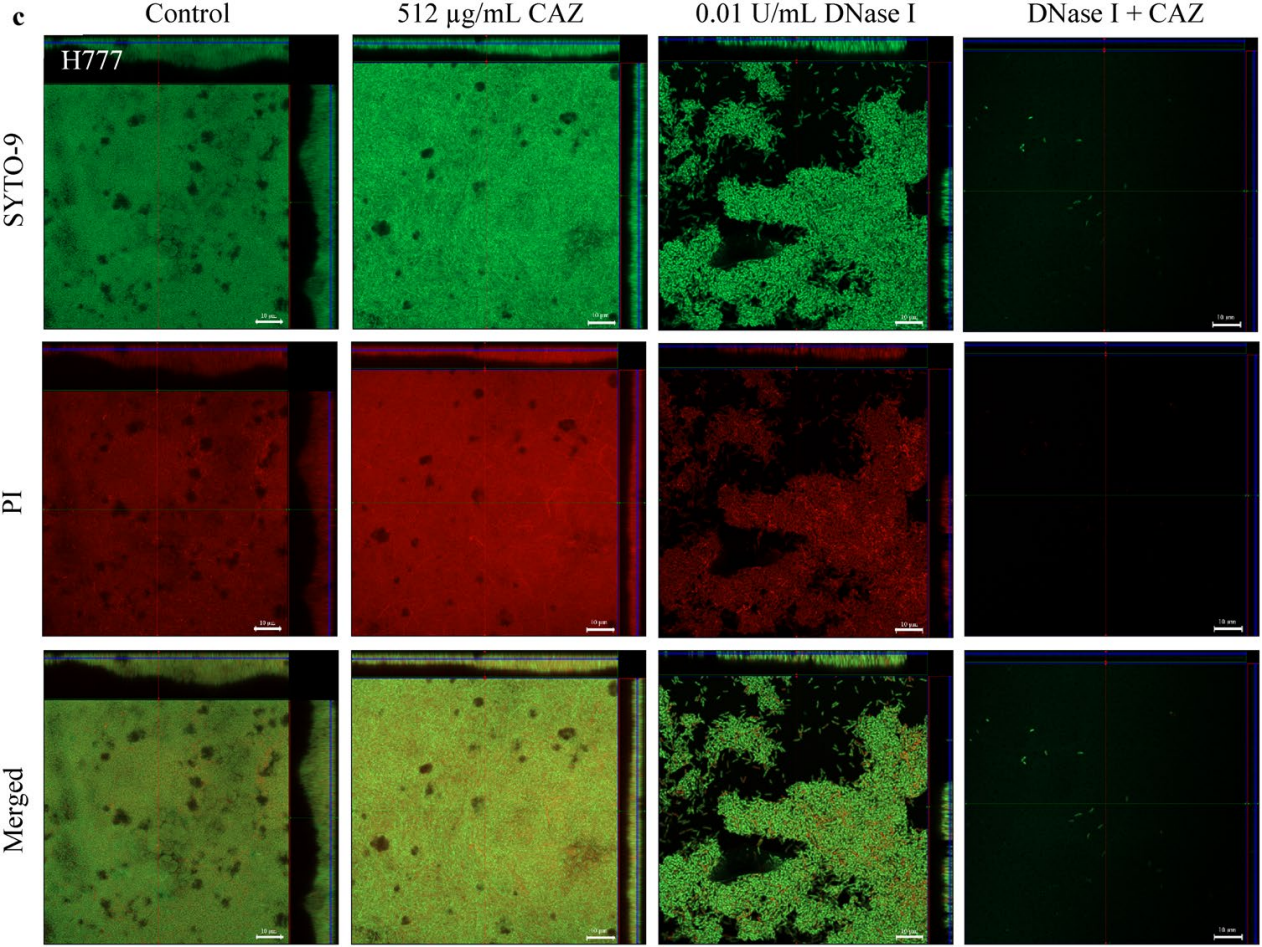
Live/dead images from CLSM of *B. pseudomallei* biofilm. The CLSM images of *B. pseudomallei* L1 (**a**), P1 (**b**) and H777 (**c**) biofilms grown statically on glass cover slips in LB broth before staining with 3.34 µM/ml SYTO-9 (live cell, green) and 5 µg/mL PI (dead, red). The biofilm was treated with LB (control), 0.01 U/ml DNase I, 512 µg/mL ceftazidime (CAZ), and DNase I combined with CAZ. These CLSM images are representatives of three independent experiments. The images were taken under a Zeiss 800 CLSM microscope (63 × magnification). Scale bar represents 10 µm.

To confirm the competence of DNase I to facilitate CAZ killing activity against embedded *B. pseudomallei* biofilm cells, live/dead staining was evaluated under CLSM. The results confirmed biofilm erosion. In conjunction with this, the live/dead ratio dropped considerably in all three *B. pseudomallei* strains compared to that of untreated controls (*p* < 0.05 in each case) (Fig. 4). Furthermore, DNase I could greatly enhance CAZ killing of *B. pseudomallei* L1 and H777 biofilm cells compared to CAZ alone (*p* < 0.001). These data emphasized that DNase I promoted CAZ efficiency leading to CAZ susceptibility of *B. pseudomallei* biofilm cells.

**Figure 5:**
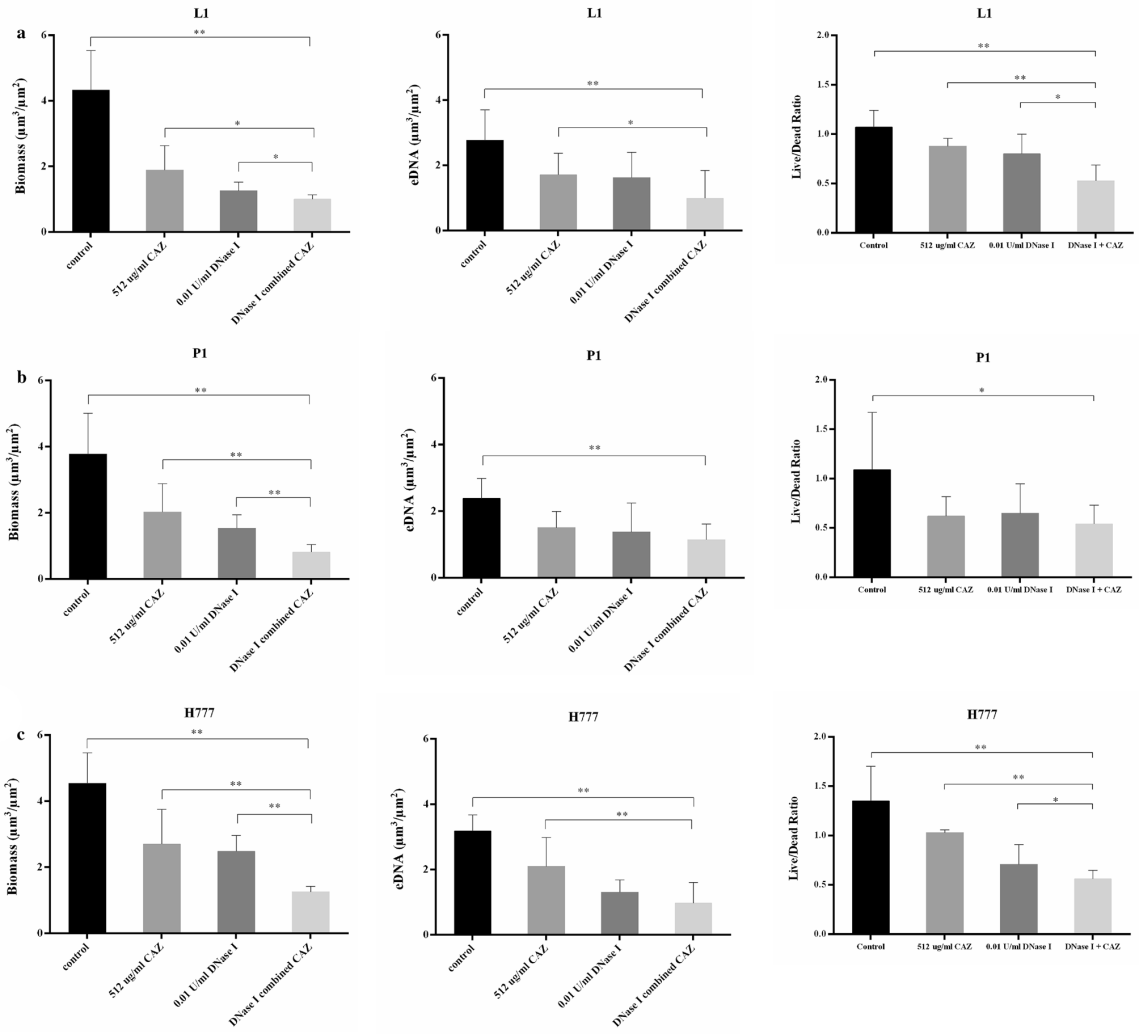
COMSTAT analysis of *B. pseudomallei* L1, P1 and H777 biofilm biomass, eDNA and Live/Dead ratio. *B. pseudomallei* L1, P1 and H777 biofilm grown in LB were treated 0.01 U/mL DNase I, 512 µg/mL ceftazidime (CAZ), and DNase I combined with CAZ. The biofilm biomass, eDNA and Live/Dead ratio of *B. pseudomallei* L1 (**a**), P1 (**b**) and H777 (**c**) were obtained from 18 CLSM images from three independent experiments using COMSTAT analysis. Statistical significance was calculated using One-way ANOVA. Asterisks indicate statistical significance as follow: *p < 0.05 and **p < 0.001.

There was a remarkable reduction in the biomass of all tested *B. pseudomallei* biofilms compared to that of untreated controls (*p* < 0.001) (72–79% reduction), and either CAZ or DNase I alone (*p* < 0.05 in each case) (Fig. 5). The eDNA in all tested *B. pseudomallei* biofilms treated with DNase I combined with CAZ was much lower than in the untreated controls (*p* < 0.001) (52–69% reduction) and less than that treated with CAZ alone in *B. pseudomallei* L1 and H777 (*p* < 0.05 and 0.001, respectively). This evidence indicated the effectiveness of DNase I to degrade eDNA leading to biofilm deterioration.

### DNase I combined with CS/CAZ effectively boosted CAZ killing ability against *B. pseudomallei* shedding planktonic and biofilm Cells

**Figure 6:**
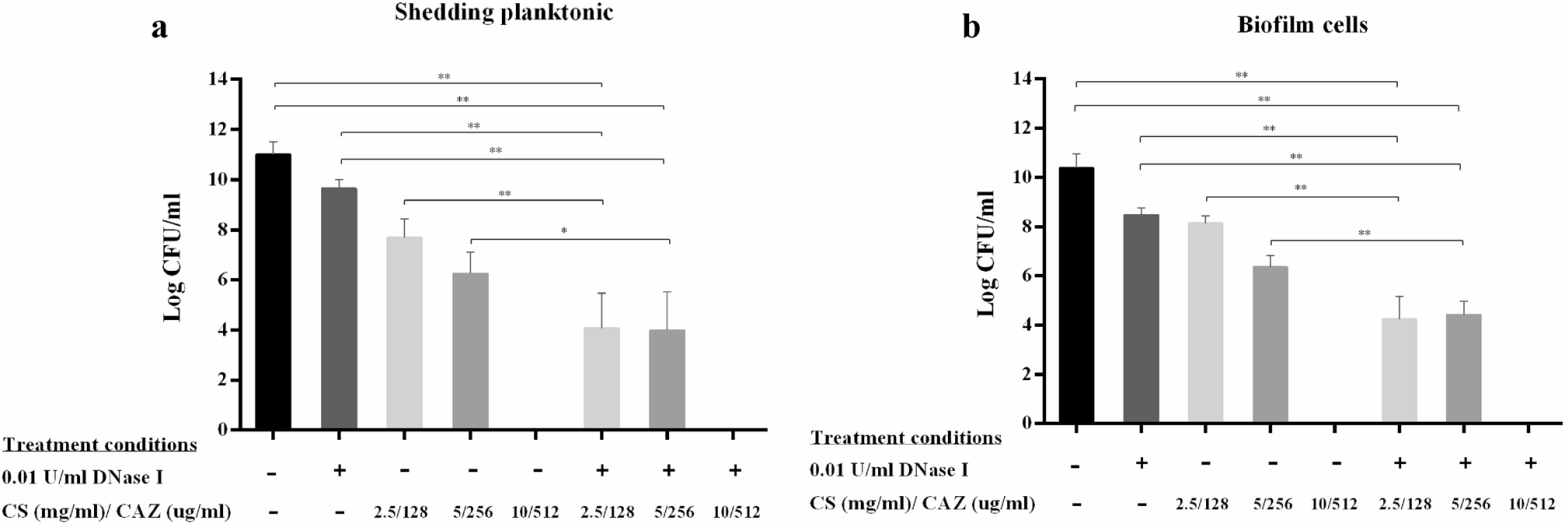
Combination of DNase I with CS/CAZ improved killing efficiency against shedding planktonic and biofilm cells of *B. pseudomallei* H777. *Burkholderia pseudomallei* H777 biofilm growth on pegs was untreated or treated with 0.01 U/mL DNase I alone for 24 h followed by addition of CS/CAZ (various concentrations) for another 24 h. The number of shed planktonic cells in the supernatant (**a**) and of biofilm cells on pegs liberated by sonication (**b**) were enumerated. The experiment was performed in duplicate in each of three independent experiments. Statistical significance was calculated using one-way ANOVA. Asterisks indicate statistical significance as follows: *p < 0.05 and **p < 0.001.

We next combined DNase I with CS/CAZ, the agent previously reported to improve bactericidal competence against *B. pseudomallei* biofilms^38^, to test their combined effect against shedding planktonic and embedded biofilm cells. DNase I (0.01 U/mL) and CS/CAZ were combined at various concentrations: 2.5 mg/mL CS/128 µg/mL CAZ, 5 mg/mL CS/256 µg/mL CAZ and 10 mg/mL CS/ 512 µg/mL CAZ. The results revealed that DNase I combined with 10 mg/mL CS/512 µg/mL CAZ completely killed all shed planktonic and biofilm cells (Fig. 6). DNase I combined with CS/CAZ at 2.5 mg/mL CS/128 µg/mL CAZ and 5 mg/mL CS/256 µg/mL CAZ significantly reduced numbers of both shedding planktonic and biofilm cells compared to untreated controls and CS/CAZ alone (*p* < 0.05 and 0.001). The most striking result to emerge from the data is that the combination of DNase I and CS/CAZ could kill both planktonic and embedded biofilm cells of *B. pseudomallei*.

### DNase I dispersed the 24-h established biofilm but failed to improve CAZ competence to kill *B. pseudomallei* biofilm cells

**Figure 7:**
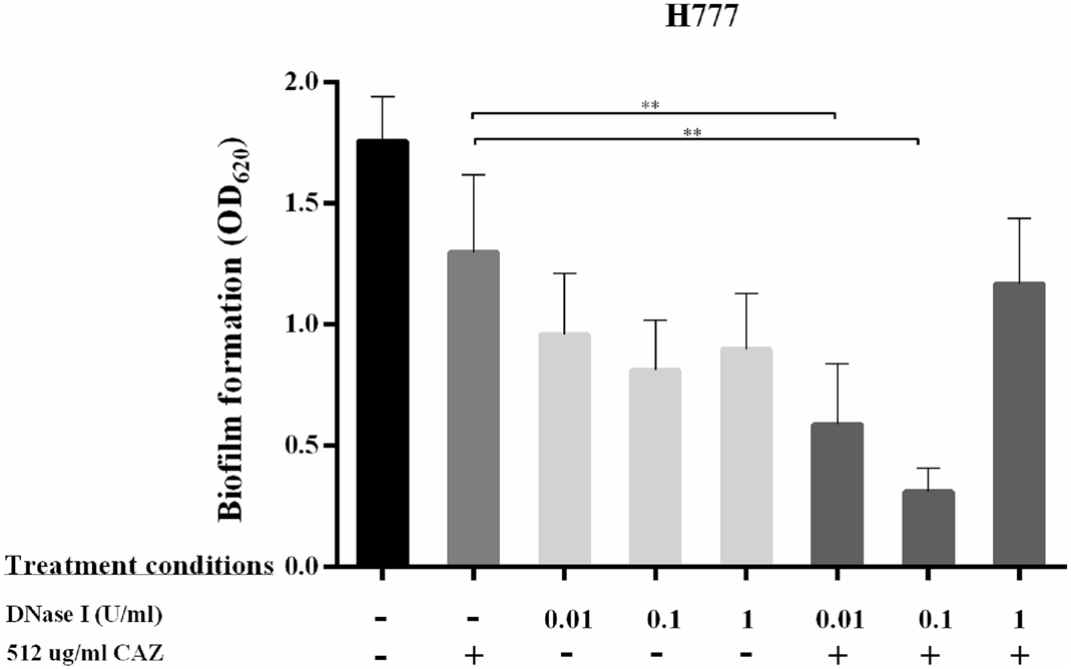
DNase I (0.01 and 0.1 U/mL) combined with CAZ dispersed the *B. pseudomallei* H777 pre-formed biofilm. The 24 h pre-formed *B. pseudomallei* H777 biofilm grown on pegs in 96-well plate was treated with 0.01, 0.1, and 1 U/mL DNase I and 512 µg/mL CAZ for another 24 h. The 2-day biofilm was examined using crystal violet staining. The experiment was performed in duplicate in each of three independent experiments. **p < 0.001.

**Figure 8:**
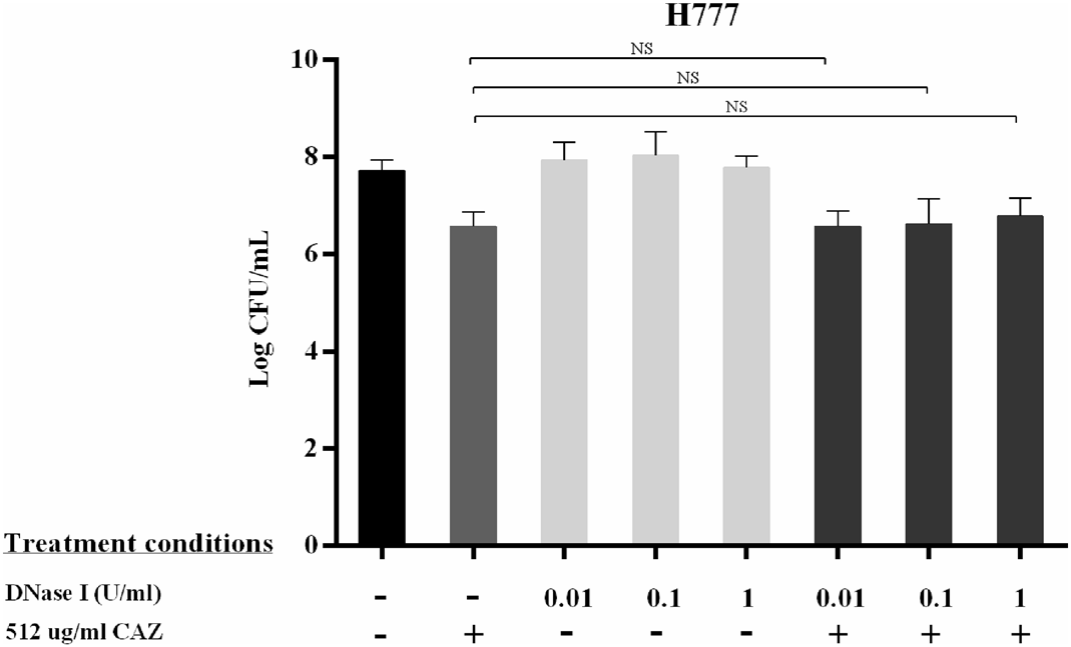
DNase I combined with CAZ failed to kill *B. pseudomallei* H777 cells in pre-formed biofilm. The 24 h pre-formed *B. pseudomallei* H777 biofilm grown on pegs in 96-well plates was treated with 0.01, 0.1 or 1 U/mL DNase I and/or 512 µg/mL CAZ for another 24 h. The 2-day viable biofilm cells were liberated by sonication for bacterial enumeration. The experiment was performed in duplicate in each of three independent experiments. NS indicates no significant difference.

We next investigated the ability of DNase I to disperse and facilitate CAZ killing of the 24-h established *B. pseudomallei* biofilm. DNase I (0.01, 0.1 or 1 U/mL) with or without 512 µg/mL CAZ was added to the pre-formed biofilm. Biofilm biomass declined significantly using 0.01 and 0.1 U/mL DNase I combined with CAZ compared to the effect of CAZ alone (*p* < 0.001) (Fig. 7). However, there was no evidence that DNase I could assist CAZ in the killing of embedded biofilm cells (Fig. 8).

## Discussion

Prior studies have noted that *Burkholderia pseudomallei* biofilm reduces antibiotic susceptibility by limiting antibiotic penetration^23,24^ and is correlated with persistent infections^18^. The eDNA, a key constituent of the *B. pseudomallei* biofilm matrix, is liberated from living biofilm cells^20^. However, the extent to which antibiotic tolerance of *B. pseudomallei* biofilm is mediated by the presence of eDNA remains to be elucidated. Additionally, bacteria within biofilms are generally more resistant to antibiotics and support the reestablishment of the biofilm construction^39^. Therefore, inhibition of *B. pseudomallei* biofilm formation, biofilm dispersion and eradication of biofilm cells are all crucial to minimize antibiotic resistance, prevent recurrence and lower mortality rates of life-threatening melioidosis. Recent research on biofilm resistance has trailed many different compounds to destroy the biofilm matrix and release the planktonic cells to restore the susceptibility to conventional antibiotics^40^. In this study, we extended our previous finding that DNase I degrades eDNA, thus inhibiting and dispersing *B. pseudomallei* biofilm^20^. We speculated that biofilm inhibition and dispersion will facilitate and enhance CAZ killing of biofilm cells. Our current results further demonstrate the ability of 0.01, 0.1 and 1 U/mL DNase I to degrade eDNA in biofilm matrix, suppress biofilm formation and, crucially, to increase CAZ susceptibility of all three clinical *B. pseudomallei* isolates. The presence of DNase I improved the susceptibility of *B. pseudomallei* biofilm cells to 512 µg/mL CAZ. This is a much lower concentration than the previously determined MBEC value for CAZ alone at 2,048 µg/mL^25^. Bactericidal activity was enhanced when 0.01 U/mL DNase I was combined with 512 µg/mL CAZ. Furthermore, the potential antibacterial and antibiofilm properties of CS/CAZ concurred with our initial finding^38^ in which CS/CAZ at 2.5 mg/mL CS/128 µg/mL CAZ and 5 mg/mL CS/256 µg/mL CAZ with 0.01 U/mL DNase I significantly improved the efficiency of CAZ to eradicate both shed planktonic and biofilm cells of *B. pseudomallei*. DNase I could inhibit formation and disrupt the biofilm matrix, allowing the antimicrobial substance to target the detached cells. These findings suggest that DNase I in combination with antimicrobial agents may be a better alternative approach against biofilm-associated pathogens to disperse biofilm and enhance bactericidal efficiency.

Extracellular DNA is a key target for dispersing biofilm and improving the vulnerability of biofilm cells to antibiotics^41^. Our results broadly support the work of other studies in this area linking Dnase I with biofilm interruption and increased susceptibility to antimicrobial agents. Li and colleagues revealed that the enzymatic activity of DNase I and dextranase could efficiently reduce biofilm adhesion and improve susceptibility of *Enterococcus faecalis* biofilms to 2% chlorhexidine^35^. Cavaliere and colleagues found that the presence of the cation chelator ethylenediaminetetra-acetic acid (EDTA) and DNase I destabilized nontypeable *Hemophilus influenzae* biofilms and enhanced susceptibility to ampicillin and ciprofloxacin^34^. Challenges for the clinical translation of biofilm-dispersing enzymes to avoid detrimental effects in vivo were explored as a novel therapeutic approach for biofilm-associated infections^42^.

The CLSM images of biofilm wiped out consistent with the drop of eDNA and live/dead ratio emphasized the potential of DNase I to degrade eDNA and facilitate CAZ bactericidal competency (Figs. 3, 4). It seems possible that these results are due to the cleavage of eDNA leads to biofilm alteration that increased antibiotic penetration and enhance the efficacy of antibiotic resulted in decrease biofilm biomass and biofilm-associated cell numbers^43^. Use of CAZ alone induced the filamentation of *B. pseudomallei* cells. The reversible filamentation induced by either sublethal concentrations of CAZ or prolonged antibiotic exposure can possibly affect antibiotic resistance^44^. Moreover, the filamentous appearance of clinical *B. pseudomallei* CAZ-resistant variants associated with treatment failure during prolonged CAZ therapy of natural infection have been demonstrated^17^. This phenomenon is caused by inhibition of cell division due to inactivation of penicillin-binding protein (PBP)-3 leading to growth into long filaments. Various CAZ concentrations inhibit PBP-3 causing filament formation in *Escherichia coli, Klebsiella pneumoniae, Pseudomonas aeruginosa* and *Acinetobacter baumannii*, suggesting additional risks during empirical treatment of severe infections^45^.

Recent research on biofilm resistance has focused on different compounds that can destroy the biofilm matrix and release planktonic cells to be attacked by conventional antibiotics^40^. Therefore, the combination of DNase I with antimicrobial agents to eradicate not only biofilm biomass but also biofilm cells and shed planktonic cells would improve the antibiotic susceptibility of biofilm-associated *B. pseudomallei* infections. We previously showed that DNase I, used in conjunction with an antibacterial and antibiofilm agent, CS/CAZ^38^, significantly improved eradication of biofilm cells and of shed planktonic cells relative to CS/CAZ alone. This observation highlights a potential novel strategy to overcome the inherent resistance of *B. pseudomallei* biofilms to antibiotics. Our results are consistent with the report of the efficacy of CS gel loaded with solid lipid nanoparticles of silver sulfadiazine supplemented with DNase I against *P. aeruginosa* biofilm in biofilm-associated wound infection^36^. Clinical studies have demonstrated the ability of recombinant human deoxyribonuclease (rhDNase) to cleave eDNA as a mucolytic agent that can improve mucociliary clearance, increase lung function and reduced the incidence of respiratory-tract infections in cystic fibrosis (CF)^46,47^. Our findings may translate to the use of DNase I combined with CAZ for better treatment of biofilm-associated *B. pseudomallei* infections.

In general, mature biofilm resists antimicrobial agents by limiting diffusion of these agents into the matrix and by containing persister cells which can survive in the presence of antibiotics^40^. The use of DNase together with CAZ against established biofilm can decrease biofilm formation but not effectively kill the established biofilm cells (Figs. 6, 7). This result may be explained by the fact that effective DNase treatment depends on the age of the biofilm. Young biofilms are simply dispersed but this is not the case for biofilm that has aged beyond a certain point^41^. This suggests that an established biofilm matrix may comprise of additional extracellular polymeric substances including polysaccharides, proteins and lipids, that provide stability^48^ and against which DNase I is less effective. DNase I treatment interfered with *Listeria monocytogenes* biofilm attachment but incompletely dispersed the established biofilm. However, the addition of proteinase K completely dispersed the biofilm. These data suggest that *L. monocytogenes* biofilm is composed of DNA and proteins^32^. Notably, the combination of trypsin and DNase I effectively function as an anti-biofilm agent against dual-species biofilms of *Staphylococcus aureus* and *Pseudomonas aeruginosa* and reduce the MBEC of antibiotics^49^. In addition, combined DNase and proteinase interfere with the composition and structural integrity of multispecies oral biofilms^50^. This may imply that a mixture of enzymes targeting components of the biofilm matrix may be utilized to disperse established biofilms: subsequent supplementation with antibiotics could then kill shedding planktonic cells. However, our preliminary results indicated that there was no significant difference in *B. pseudomallei* H777 biofilm inhibition or dispersion between untreated controls and treatments using proteinase K. Further work may be required to verify the components of *B. pseudomallei* biofilm.

Overall, DNase I combined with antimicrobial agents could have a great impact on future clinical treatments to prevent biofilm formation, especially for *B. pseudomallei.* Despite these promising results, questions remain. A note of caution is due here since the high concentration of CAZ may cause difficulties for clinical management. Further studies are required to optimize clinical achievable CAZ concentration and investigate the synergistic activity of DNase I and antimicrobial agents against mature *B. pseudomallei* biofilm. Thus, the potency of synergistic combinations of DNase I with antimicrobial CS and the drug of choice to treat melioidosis, CAZ, has potential for melioidosis management.

## Conclusions

The present study was designed to determine the contribution of eDNA to antibiotic resistance by *B. pseudomallei* using DNase I. The most obvious finding to emerge from this study is that DNase I degraded eDNA leading to biofilm inhibition and enhanced CAZ efficacy resulted in a 3–4 log reduction in viable *B. pseudomallei* biofilm cell numbers. The combination of *B. pseudomallei* shedding planktonic and biofilm cells DNase I with CS/CAZ completely eradicated *B. pseudomallei* shedding planktonic and biofilm cells. The findings of this study provide a potential therapeutic approach to improve effectiveness treatment against *B. pseudomallei* biofilm associated infections.

## Materials and methods

### Ethics statement

*Burkholderia pseudomallei* clinical isolates H777, P1 and L1 (from the Melioidosis Research Center, Khon Kaen University (MRC, KKU) were used. These isolates had been collected as a part of a study of the epidemiology of *B. pseudomallei* approved by the Khon Kaen University Ethics Committee for Human Research (HE490324). Patients cannot be identified as the isolates de-identified when we received them. All methods were performed in accordance with the relevant guidelines and regulations.

### *Burkholderia pseudomallei* strains and growth conditions

*Burkholderia pseudomallei* H777, L1 and P1 from glycerol stock at -80 °C were grown on Ashdown’s agar and incubated at 37 °C for 48 h. Inoculum culture was prepared from a single colony of *B. pseudomallei* in 3 mL of Luria–Bertani (LB) broth and incubated at 37 °C with shaking (200 rpm) for 18–20 h. Thereafter, 2% inoculum was inoculated into fresh LB. The bacterial culture was adjusted to 10^7^ or 10^8^ CFU/mL as the starter inoculum^19,20^.

### Ceftazidime (CAZ) preparation

Ceftazidime hydrate (Sigma-Aldrich, St. Louis, MO, USA) was dissolved in sterile injected water before filter sterilization. CAZ stock was aliquoted and stored at -20 °C until used.

### Chitosan-linked ceftazidime (CS/CAZ) preparation

Chitosan from shrimp shells with ≥ 75% deacetylation (Product number C3646, Sigma-Aldrich, Saint Louis, Missouri, USA) (CS) linked to CAZ (CS/CAZ) was prepared as previously described^38^. In brief, 20 mg/mL of CS stock solution was dissolved in 1% v/v acetic acid at 160 rpm overnight at room temperature. The stock solution was adjusted to pH 5.6 before being autoclaved at 121 °C for 20 min and stored at 4 °C until used. The sterile 5000 µg/mL CAZ stock was added dropwise into 20 mg/mL of CS with continuous magnetic stirring at 160 rpm for 24 h to obtain the stock CS/CAZ of 20 mg/mL of CS/1,024 µg/mL CAZ. The solution was used immediately or stored at 4 °C and used within 7 days. On the day of the experiment, the CS/CAZ stock solution was twofold serially diluted to the designated concentration.

### Biofilm inhibition and dispersal determination

*Burkholderia pseudomallei* biofilm was quantified as a 2-day biofilm on polystyrene peg lids (Nunclon™, Roskilde, Denmark) using crystal violet as previously described^51^ with slight modification. Briefly, 200 µL of each bacterial starter culture (10^7^ CFU/mL) was inoculated into duplicate wells of a 96-well plate as untreated controls. For biofilm-inhibition experiments requiring treatment with DNase I throughout 48 h, 180 µL of bacterial starter was cultured in the presence of 20 µL of DNase I (Roche, Mannheim, Germany) at final concentrations of 0.01, 0.1 and 1 U/mL in DNase I buffer (400 mM Tris–HCl, 100 mM NaCl, 60 mM MgCl_2_·6H_2_O, and 10 mM CaCl_2_·2H_2_O). Thereafter, the pegs were immersed into the mixture and incubated at 37 ºC for 24 h. The 24-h preformed biofilms on the pegs were rinsed with sterile phosphate buffer saline (PBS), pH 7.4 for 1 min to remove unattached planktonic cells then immersed in a new well plate containing fresh LB broth with either the same DNase I concentration, CAZ at 512 µg/mL (the sub concentration of minimum biofilm eradication concentration (MBEC)^25,51^ or DNase I combined with CAZ for another 24 h at 37 ºC to obtain 2-day biofilm. Thereafter, the biofilms on pegs were rinsed once with sterile PBS for 1 min, fixed with 99% methanol for 15 min and stained with 2% w/v crystal violet for 5 min. The excess stain was removed using running tap water and air-dried, the crystal violet stain on each peg was dissolved by immersion into 200 µL 33% (v/v) glacial acetic acid and the optical density measured at 620 nm using a microplate reader (TECAN Safire, Port Melbourne, Australia).

For the biofilm dispersal experiments, the 24-h established biofilm in LB broth on pegs was exposed to DNase I and CAZ for another 24 h. The treated 2-day biofilm was then examined by crystal violet staining as above.

### *Burkholderia pseudomallei* biofilm cells enumeration

To enumerate viable *B. pseudomallei* biofilm cells from biofilm inhibition and dispersion experiments, the 2-day biofilms on pegs were rinsed with sterile PBS for 1 min. Thereafter, each peg lid was transferred to a new 96 well plate contained 200 µl Muller Hilton broth and sonicated for 5 min to liberate biofilm cells. Subsequently, the bacterial suspension was serially diluted for bacterial enumeration using the drop plate technique on LB agar and incubated at 37 ºC for 24 h and reported as CFU/mL^52^.

### Confocal laser scanning microscope (CLSM) observation

To assess the impact of DNase I on CAZ efficacy in the biofilm inhibition experiment, *B. pseudomallei* biofilm structure and eDNA were observed on sterile 12 mm-diameter round glass coverslips held by an Amsterdam Active Attachment (AAA) model with slight modifications from a previously described method^19,51^. In brief, 1 mL of bacterial starter culture (10^8^ CFU/mL) in LB medium and 0, 0.01, 0.1 or 1 U/mL DNase I was added to each well of a 24-well plate (Costar® #3524, Corning, NY, USA). The coverslips were allowed to develop biofilm at 37 °C for 24 h. The coverslips were then washed once with sterile PBS, pH 7.4 and further incubated in fresh LB medium with DNase I, 512 µg/mL CAZ or the mixture of both agents for another 24 h. The 2-day biofilms on the coverslips were rinsed three times with sterile PBS prior to staining with 50 µg/mL fluorescein isothiocyanate-concanavalin A (FITC-Con A) (Sigma-Aldrich, Saint Louis, Missouri, USA). FITC-ConA binds α − D-mannose or α − D-glucose that are present in various sugars, glycoproteins and glycolipids including microbial cell walls (representing biofilm biomass, green) and 2 µM TOTO-3 (Thermo fisher Scientific, Oregon, USA), which binds eDNA (red) for 20 min. Separately, the viability of biofilm cells was examined using 3.34 µM/mL SYTO 9 and 5 µg/mL propidium iodide (PI) (Invitrogen, Thermo fisher Scientific, Oregon, USA) staining for 15 min. The biofilms were subsequently fixed with 2.5% glutaraldehyde in PBS for 3 h before washing with sterile PBS 3 times and air-dried for 24 h at room temperature. The biofilm structure and eDNA were visualized under a confocal laser scanning microscope (CLSM, LSM 800, Carl Zeiss, Jena, Germany). The excitation/emission maxima for these dyes were approximately 495/519 nm for FITC-ConA, 261/661 nm for TOTO-3, 483/500 nm for SYTO 9 and 305/617 nm for PI. The biofilm intensity was analyzed by z-stack processing using Zen blue software^19,53^. Biomass of adherent cells and eDNA quantity were calculated from 18 CLSM images using the COMSTAT computer program^54^. The biofilm cell viability was presented as live/dead ratio.

### Statistical analysis

Statistical analyses were performed using SPSS software, version 23 (SPSS Inc., Chicago, IL, USA). Data were analyzed for statistical significance using the one-way ANOVA followed by Tukey post-hoc test, or Games-Howell post-hoc test to correct for variance heterogeneity. The levels required for statistical significance were *p < 0.05 and **p < 0.001.

## Data Availability

The datasets used and/or analyzed during the current study available from the corresponding author on reasonable request.
